# Supplementary material for: Seabird colonies as important global drivers in the nitrogen and phosphorus cycles
Source: Nat Commun. 2018 Jan 23;9:246. doi: 10.1038/s41467-017-02446-8 (PMC5780392; doi:10.1038/s41467-017-02446-8)
Supplement: Supplementary file 1 — Supplementary Information [file 41467_2017_2446_MOESM1_ESM.pdf]

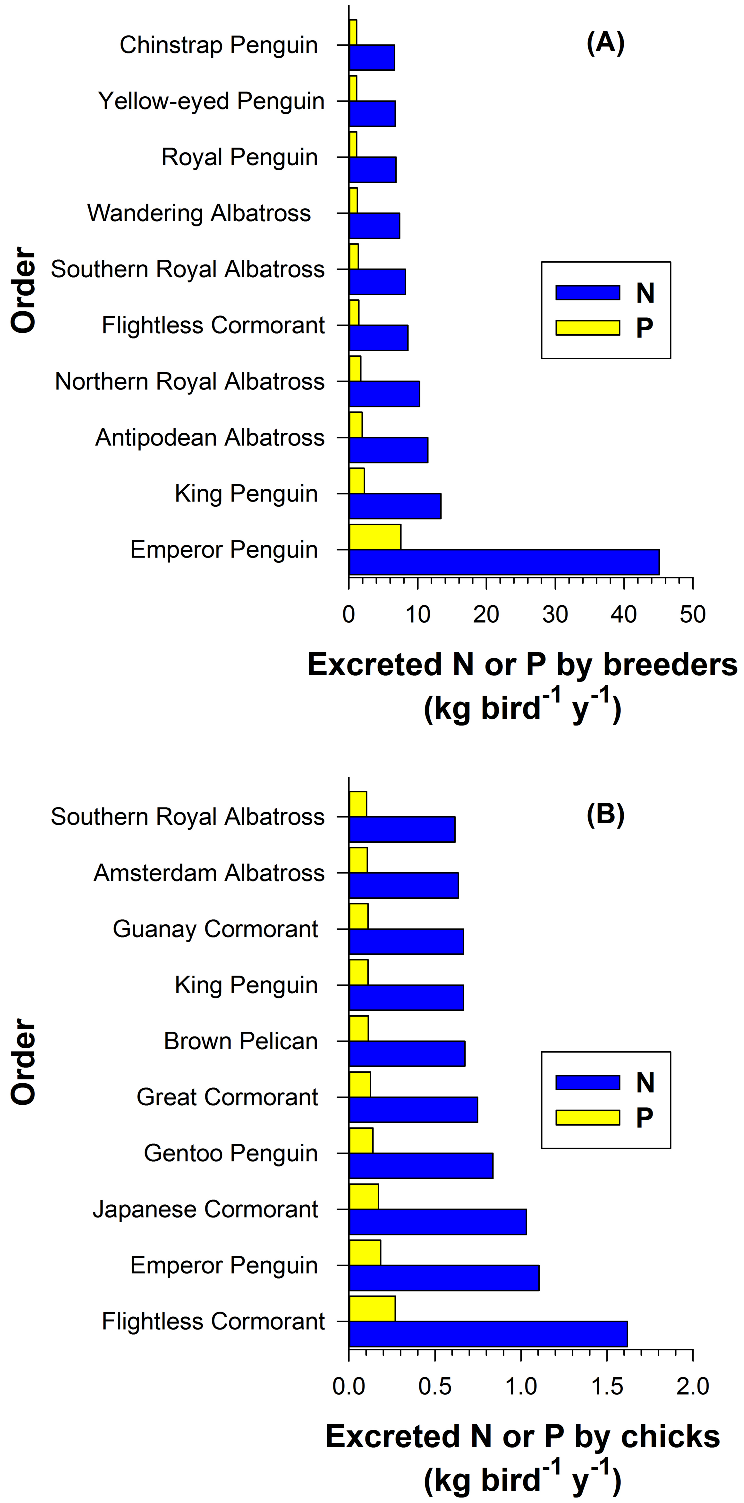

Supplementary Figure 1. Excretion of N and P per seabird: (A) adults, and (B) chicks.

Supplementary Table 1. Size of nitrogen major compartments/reservoirs and fluxes between them.

Compartment/reservoir	Mass of N (Ggx10^9)	Reference
Atmospheric N_2	3950	15; 5
Nitrogen in sedimentary rocks	0.9996	9
Ocean:		5
N_2	20	
NO_3^-	0.57	
Organic soils	0.19	9
Land biota	0.00010	9
Marine biota	0.00050	9
Nitrogen sequestered in ice	0.000000260	21
**Compartment/reservoir**	**Fluxes of N (Gg y^{-1}x10^3)**	**Reference**
Atmospheric N_2 fixed by biological process	58	18; 4
Nitrogen fixation in oceans	140	20
Lightning	5	4
Nitrogen fixation by agricultural crops	50-70	6
Legume cultivation	40	1
Rice cultivation	5.0	1
Nitrogen buried in deep ocean sediments	20	20
N_2O emissions from ocean to atmosphere	0.5-5.5	19; 4
Nitrogen input into the ocean by rivers	66	14
Nitrogen input into the ocean by ground water	4	19
Atmospheric deposition on ocean	8.4	5
N transfer from surface ocean to land via fishing activities	3.7	10
Ornitheutrofication. Transfer of N from surface ocean to land by seabirds. Total N:		This study
Breeding seabirds	0.60	
Total population	3.80	
Labile (leachable)	0.07	

Supplementary Table 2. Major compartments/reservoirs of phosphorous in the Earth's surface and fluxes between the different compartments (adapted from 12).

Compartment/reservoir	Mass of P ($Ggx10^6$)	Reference
Land soil (soil<60 cm depth)	96.1-206	8; 13
Land biota	2.6-3.0	8; 13
Surface ocean (<300m depth)	2.6	8; 13
Oceanic biota	0.050-0.138	8; 13; 7; 9
Atmospheric P	0.00003	13; 7; 9
Domestic animals	0.0323	Present study using data from 3 and 17
**Compartment/reservoir**	**Fluxes of P ($Gg\ P\ y^{-1}x10^3$)**	**Reference**
Rock/sediment → soil (weathering, soil accumulation)	20	11; 9
Soil → rock/sediment (deep burial, lithification)	9.3-19	8; 9
Soil → land biota	62-200	8; 7
Soil → surface ocean (present-day river dissolved P):		
Dissolved inorganic P	0.8-1.4	2; 12
Dissolved organic P	0.2	
Soil → surface ocean (present-day total river P)	17.3-20.1	2; 12
Atmosphere → land soil	3.10	9
Atmosphere → surface ocean	0.60 - 1.50	13; 7
Surface ocean → atmosphere	0.31	13; 7
Surface ocean → land (fisheries)	0.32	9
Orniteutrophication.Transfer of P from surface ocean to land by seabirds. Total P:		This study
Breeding seabirds	0.10	
Total population	0.63	
Labile (leachable) P	0.02	

Supplementary Table 3. Mean concentrations of total and labile (soluble) N and P (mg kg^{-1}) in seabird faecal materials.

Element	Charadriiformes (mg kg^{-1})	Reference	Pelecaniformes (mg kg^{-1})	Reference	Procellariiformes (mg kg^{-1})	Reference	Sphenisciformes (mg kg^{-1})	Reference
Total N	67708	2,3,4,5,6,7,8,9	105400	4	175833	13,14	113781	3,16,17,19
Labile N	5715	2,6,7,8,9	13340	*	31860	13	12982	16,17
Total P	24782	2,4,5,6,7,8,9,10,11	92933	4	17600	13,14,15	45244	19,20,21
Labile P	6613	7,8,9,10,11	15000	10	3768	*	9685	*

Labile N comprises NO_3^- and NH_4^+ soluble in water or neutral salts (e.g., KCl or $MgCl_2$ 1M), whereas labile P includes also the phosphate soluble in Mehlich 3 extractant (0.2N CH_3COOH-0.25N NH_4NO_3-0.015N NH_4F-0.013N HNO_3-0.001M EDTA). *No data available for the N and P labile forms. For these particular cases, the labile forms of N and P were calculated based on average labile values of these two elements that were applied to the total seabird population.

References

1. Canfield, D., Thamdrup, B. & Kristensen, E. *Aquatic Geomicrobiology*. Advances in Marine Biology Vol. 48. (Elsevier, Amsterdam, The Netherlands, 2005).
2. Compton, J. et al. in *Marine Authigenesis: From Global to Microbial* (eds Glenn, C. R., Prévôt-Lucas, L. & Lucas, J.) 21-33 (SEPM Special Publication Nº 66, 2000).
3. FAOSTAT, Food and agriculture organization of the United Nations, Statistical Database, (http://faostat,fao,org, 2015).
4. Fowler, D. et al. The global nitrogen cycle in the twenty-first century. *Phil. Trans. R. Soc. B* **368,** 20130164 (2013).
5. Galloway, J. N. et al. The nitrogen cascade. *BioScience* **53**, 341-356 (2003).
6. Herridge, D. F., Peoples, M. B. & Boddey, R. M. Global inputs of biological nitrogen fixation in agricultural systems. *Plant Soil* **311**, 1-18 (2008).
7. Jahnke, R. A. in *Global Biogeochemical Cycles*. (eds Butcher, S. S., Charlson, R. J., Orians, G. H., & Wolff, G. V.) 301-315. (Academic Press, London, UK, 1992).
8. Lerman, A., Mackenzie, F. T. & Garrels, R. M. Modeling of geochemical cycles: phosphorus as an example. *Geol. Soc. Am. Memoirs* **142**, 205-217 (1975).
9. Mackenzie, F.T. et al. in *Interactions of C, N, P and S Biogeochemical Cycles and Global Change* (eds Wollast, R., Mackenzie, F. T., & Chou, L.) vol. 4. 1-61 (Springer-Verlag, Berlin Heidelberg,1993).
10. Maranger, R., Caraco, N., Duhamel, J. & Amyot, M. Nitrogen transfer from sea to land via commercial fisheries. *Nat. Geosci.* **2**, 111-113 (2008).
11. Meybeck, M. Carbon, nitrogen, and phosphorus transport by world rivers. *Am. J. Sci.* **282**, 401-450 (1982).
12. Paytan, A. & McLaughlin, K. The oceanic phosphorous cycle. *Chem. Rev*. **107**, 563-576 (2007).
13. Richey, J. E. in *The Major Biogeochemical Cycles and Their Interactions* (eds Bolin, B. & Cook, R. B.) 51-56 (John Wiley & Sons, Chichester, UK, 1983).
14. Ruttenberg, K. C. in *Biogeochemistry* (ed Schlesinger, W. H.) Vol. 8, 585-643, *Treatise on Geochemistry* (eds Holland, H. D. & Turekian, K. K.) (Elsevier-Pergamon, Oxford, 2003).
15. Schlesinger, W. *Biogeochemistry: An Analysis of Global Change*. (Academic Press, Amsterdam, The Netherlands, 1997).
16. Seitzinger, S. P., Harrison, J. A., Dumont, E., Beusen, A. H. W & Bouwman, A. F. Sources and delivery of carbon, nitrogen, and phosphorus to the coastal zone: An overview of Global Nutrient Export from Watersheds (NEWS) models and their application. *Global Biogeochem. Cycles* **19**, gb4s01, doi:10.1029/2005gb002606 (2005).
17. Sheldrick, W., Syers, J. K. & Lingard, J. Contribution of livestock excreta to nutrient balances. *Nutr. Cycling Agroecosyst.* **66**, 119-131 (2003).
18. Vitousek, P. M., Menge, D. N. L., Reed, S. C. & Cleveland, C. C. Biological nitrogen fixation: rates, patterns, and ecological controls in terrestrial ecosystems. *Phil. Trans. R. Soc. B* **368**, 20130119 (2013).

19. Voss, M. et al. in *The European Nitrogen Assessment* (eds Sutton, M. A. et al.) 147-176 (Cambridge University Press, New York, 2011).
20. Voss, M. et al. The marine nitrogen cycle: recent discoveries, uncertainties and the potential relevance of climate change. *Phil. Trans. R. Soc. B* **368**, 20130121 (2013).
21. Wolff, E. W. Ice sheets and nitrogen. *Phil.Trans. R. Soc. B* **368**, 20130127 (2013).
22. Mehlich, A. Mehlich 3 soil test extractant: a modification of the mehlich 2 extractant. Commun. *Soil Sci. Plant Anal.* **15**, 1409-1416 (1984).
23. Sobey, G. G. & Kenworthy, J. B. The relationship between herring gulls and the vegetation of their breeding colonies. *J. Ecol.* **67**, 469-496 (1979).
24. Mizutani, H. & Wada, E. Nitrogen and Carbon Isotope Ratios in Seabird Rookeries and their Ecological Implications. *Ecology* **69 (2)**, 340-349 (1988).
25. Hahn, S., Bauer, S. & Klaassen, M. Estimating the contribution of carnivorous waterbirds to nutrient loading in freshwater habitats. *Freshw. Biol.* **52**, 2421-2433 (2007).
26. Otero, X. L. & Mouriño, J. Nitrógeno ($NH4^+$, $NO3^-$), Fósforo asimilable y metales traza (Hg, Cd, Pb, Zn, Ni y Cu) en suelos de la colonia de la gaviota patiamarilla (*Larus cachinnans*) en el Parque natural de las Islas Cíes. *Cuad. Soc. Esp. Cien. For.* **14**, 143-149 (2002).
27. Smith, J. S. & Johnson, C. R. Nutrient inputs from seabirds and humans on a populated coral cay. *Mar. Ecol. Prog. Ser.* **124**, 189-200 (1995).
28. Otero, X. L. et al. Phosphorus in seagull colonies and the effect on the habitats. The case of yellow-legged gulls (*Larus michahellis*) in the Atlantic Islands National Park (Galicia-NW Spain). *Sci. Total Environ.* **532**, 383-397 (2015).
29. Otero, X. L. *Monitorización de los procesos biogeoquímicos en la colonia de Larus cachinnans del Parque Nacional de las Islas Atlánticas*. Organismo Autónomo Parques Nacionales. Ministerio de Agricultura, Pesca, Alimentación y Medio Ambiente. Madrid, Unpublished report (2016).
30. Loder III, T. C., Ganning, B. & Love, J. A. Ammonia nitrogen dynamics in coastal rockpools affected by gull guano. *J. Exp. Mar. Biol. Ecol.* **196**,113-129 (1996).
31. Zhu, R. B. et al. Greenhouse gas emissions from penguin guanos and ornithogenic soils in coastal Antarctica: effects of freezing–thawing cycles. *Atmos. Environ.* **43**, 2336-2347 (2009).
32. Yan, H., Yuhong, W., Wenhan, C. & Liguang, S. Millennial mercury records derived from ornithogenic sediment on Dongdao Island, South China Sea. *J. Environ. Sci.* **23**, 1415-1423 (2011).
33. Fugler, S. R. Chemical composition of guano of burrowing petrel chicks (*Procellariidae*) at Marion Island. in *Antarctic Nutrient Cycles and Food Webs* (eds Siegfried, W. R., Condy, P. R. & Laws, R. M.) 169-172 (Springer-Verlag, Berlin Heidelberg, 1985).
34. Bancroft, W. J., Garkaklis, M. J. & Roberts, J. D. Burrow building in seabird colonies: a soil-forming process in island ecosystems. *Pedobiologia* **49**, 149-165 (2005).
35. Brimble, S. K. et al. High arctic ponds receiving biotransported nutrients from a nearby seabird colony are also subject to potentially toxic loadings of arsenic, cadmium and zinc. *Environ. Toxicol. Chem.* **28**, 2426-2433 (2009).

36. Zhu, R., Sun, J., Liu, Y., Gong, Z. & Sun, L. Potential ammonia emissions from penguin guano, ornithogenic soils and seal colony soils in coastal Antarctica: effects of freezing-thawing cycles and selected environmental variables. *Antarct. Sci.* **23 (1)**, 78-92 (2011).
37. Lindeboom, H. J. The nitrogen pathway in a penguin rookery. *Ecology* **65**, 269-277 (1984).
38. Mizutani, H., Kabaya, Y. & Wada, E. Ammonia volatilization and high 15N/14N ratio in a penguin rookery in Antarctica. *Geochem. J.* **19**, 323-327 (1985).
39. Zhu, R., Yashu, L., Jianjun, S., Liguang, S. & Jinju, G. Stimulation of gaseous phosphine production from Antarctic seabird guanos and ornithogenic soils. *J. Environ. Sci.* **21**, 150–154 (2009).
40. Metcheva, R., Yurukova, L. & Teodorova, S. E. Biogenic and toxic elements in feathers, eggs, and excreta of Gentoo penguin (*Pygoscelis papua ellsworthii*) in the Antarctic. *Environ. Monit. Assess.* **182**, 571-585 (2011).
41. Xianyan, Q. et al. From sea to land: assessment of the bio-transport of phosphorus by penguins in Antarctica. *Chin. J. Oceanol. Limnol.* **1**, 148–154 (2014).
42. Zhu, R. et al. Tropospheric Phosphine and Its Sources in Coastal Antarctica. *Environ. Sci. Technol.* **40**, 7656-7661 (2006).
