# Supplementary material for: Seabird colonies as important global drivers in the nitrogen and phosphorus cycles
Source: Nat Commun. 2018 Jan 23;9:246. doi: 10.1038/s41467-017-02446-8 (PMC5780392; doi:10.1038/s41467-017-02446-8)
Supplement: Supplementary file 3 — Description of Additional Supplementary Files [file 41467_2017_2446_MOESM3_ESM.pdf]

Description of Additional Supplementary Files

File Name: Supplementary Data 1
Description: Size of the worldwide seabird population arranged by species, with their corresponding amounts of excreted N and P.

File Name: Supplementary Data 2
Description: Geographic coordinates with their corresponding N and P excreted every year.
