# Supplementary material for: Seabird colonies as important global drivers in the nitrogen and phosphorus cycles
Source: Nat Commun. 2018 Jan 23;9:246. doi: 10.1038/s41467-017-02446-8 (PMC5780392; doi:10.1038/s41467-017-02446-8)
Supplement: Supplementary file 4 — Supplementary Data 1 [file 41467_2017_2446_MOESM4_ESM.docx]

**Supplementary Data 1**. Size of the worldwide seabird population arranged by species, with their corresponding amounts of excreted N and P.

| **Scientific Name** | **Common name** | **Order*** | **Breeding**  **population** | **Population of**  **breeding and**  **chick**  **seabirds**** | **Total**  **excreted N**  **(Gg N y^-1^ )***** | **Total**  **excreted P**  **(Gg P y^-1^)***** | **References** |
| --- | --- | --- | --- | --- | --- | --- | --- |
| *Aethia cristatella* | Crested Auklet | C | 8,200,000 | 10,250,000 | 2.862 | 0.477 | (1; 2; 11; 9) |
| *Aethia psittacula* | Parakeet Auklet | C | 1,200,000 | 1,500,000 | 0.211 | 0.035 | (1; 2; 11; 9) |
| *Aethia pusilla* | Least Auklet | C | 24,000,000 | 30,000,000 | 3.356 | 0.559 | (2; 11; 9) |
| *Aethia pygmaea* | Whiskered Auklet | C | 100,000 | 125,000 | 0.018 | 0.003 | (2; 11; 9) |
| *Alca torda* | Razorbill | C | 25,200 | 32,760 | 0.023 | 0.004 | (1; 2; 21) |
| *Alle alle* | Little Auk | C | 26,000,000 | 32,500,000 | 5.902 | 0.984 | (2; 11; 9) |
| *Anous minutus* | Black Noddy | C | 1,069,000 | 1,336,250 | 0.204 | 0.034 | (2; 31; 9) |
| *Anous stolidus* | Brown Noddy | C | 640,000 | 800,000 | 0.182 | 0.030 | (2; 31; 9) |
| *Anous tenuirostris* | Lesser Noddy | C | 1,200,000 | 1,536,000 | 0.200 | 0.033 | (2; 31; 9) |
| *Aphrodroma brevirostris* | Kerguelen Petrel | Pr | 1,000,000 | 1,500,000 | 0.073 | 0.012 | (2; 4; 9) |
| *Aptenodytes forsteri* | Emperor Penguin | S | 476,000 | 621,180 | 21.631 | 3.605 | (2; 9; 9) |
| *Aptenodytes patagonicus* | King Penguin | S | 4,460,000 | 5,329,700 | 60.073 | 10.012 | (2; 27; 9) |
| *Ardenna bulleri* | Buller's Shearwater | Pr | 2,500,000 | 3,000,000 | 0.212 | 0.035 | (2; 24; 9) |
| *Ardenna carneipes* | Flesh-footed Shearwater | Pr | 650,000 | 802,750 | 0.160 | 0.027 | (2; 4; 9) |
| *Ardenna creatopus* | Pink-footed shearwater | Pr | 66,667 | 83,333 | 0.013 | 0.002 | (2; 9) |
| *Ardenna gravis* | Great Shearwater | Pr | 15,000,000 | 19,950,000 | 5.641 | 0.940 | (2; 4; 9) |
| *Ardenna grisea* | Sooty Shearwater | Pr | 20,000,000 | 25,500,000 | 2.953 | 0.492 | (2; 4; 9) |
| *Ardenna pacifica* | Wedge-tailed Shearwater | Pr | 5,200,000 | 6,370,000 | 0.435 | 0.072 | (2; 4; 9) |
| *Ardenna tenuirostris* | Short-tailed Shearwater | Pr | 23,000,000 | 29,900,000 | 21.842 | 3.640 | (2; 4; 9) |
| *Brachyramphus brevirostris* | Kittlitz's Murrelet | C | 43,500 | 47,850 | 0.013 | 0.002 | (2; 30; 9) |
| *Brachyramphus marmoratus* | Marbled Murrelet | C | 256,667 | 320,833 | 0.109 | 0.018 | (2; 8; 9) |
| *Brachyramphus perdix* | Long-billed Murrelet | C | 329,667 | 412,083 | 0.125 | 0.021 | (2; 20; 9) |
| *Bulweria bulwerii* | Bulwer’s Petrel | Pr | 750,000 | 915,000 | 0.141 | 0.023 | (2; 4; 9) |
| *Bulweria fallax* | Jouanin's Petrel | Pr | 6,375 | 7,809 | 0.002 | 0.000 | (2; 4; 9) |
| *Calonectris diomedea* | Cory's Shearwater | Pr | 365,500 | 456,875 | 0.280 | 0.047 | (2; 9) |
| *Calonectris edwardsii* | Cape Verde Shearwater | Pr | 20,000 | 25,000 | 0.015 | 0.003 | (2; 9) |
| *Calonectris leucomelas* | Streaked Shearwater | Pr | 3,000,000 | 3,600,000 | 1.284 | 0.214 | (2; 4; 9) |
| *Catharacta antarctica* | Southern Skua | C | 27,000 | 43,200 | 0.063 | 0.011 | (2; 11; 9) |
| *Catharacta chilensis* | Chilean Skua | C | 6,000 | 9,000 | 0.009 | 0.002 | (2; 7; 9) |
| *Catharacta lonnbergi* | Brown Skua | C | 7,000 | 11,200 | 0.016 | 0.003 | (13; 9) |
| *Catharacta maccormicki* | South Polar Skua | C | 15,750 | 19,688 | 0.030 | 0.005 | (2; 9) |
| *Catharacta skua* | Great Skua | C | 48,000 | 66,720 | 0.079 | 0.013 | (2; 9) |
| *Cepphus carbo* | Spectacled Guillemot | C | 144,000 | 218,160 | 0.059 | 0.010 | (2; 11; 9) |
| *Cepphus columba* | Pigeon Guillemot | C | 470,000 | 662,700 | 0.135 | 0.022 | (2; 11; 9) |
| *Cepphus grylle* | Black Guillemot | C | 550,000 | 852,500 | 0.176 | 0.029 | (2; 11; 9) |
| *Cerorhinca monocerata* | Rhinoceros Auklet | C | 1,300,000 | 1,885,000 | 0.472 | 0.079 | (2; 11; 9) |
| *Chionis albus* | Snowy Sheathbill | C | 13,333 | 16,667 | 0.028 | 0.005 | (2; 15; 9) |
| *Chionis minor* | Black-faced Sheathbill | C | 10,850 | 13,563 | 0.023 | 0.004 | (2; 9) |
| *Chlidonias albostriatus* | Black-fronted Tern | C | 6,250 | 7,124 | 0.001 | 0.000 | (2; 9) |
| *Chlidonias hybrida* | Whiskered Tern | C | 600,000 | 876,000 | 0.094 | 0.016 | (2; 31; 9) |
| *Chlidonias leucopterus* | White-winged Tern | C | 2,333,333 | 3,406,667 | 0.364 | 0.061 | (2; 31; 9) |
| *Chlidonias niger* | Black Tern | C | 850,000 | 1,232,500 | 0.097 | 0.016 | (2; 31; 9) |
| *Creagrus furcatus* | Swallow-tailed Gull | C | 35,000 | 41,125 | 0.032 | 0.005 | (2; 31; 9) |
| *Daption capense* | Cape Petrel | Pr | 2,000,000 | 2,500,000 | 0.855 | 0.142 | (2; 4; 9) |
| *Daption capense australe* | Snares Cape Pigeon | Pr | 5,000 | 6,250 | 0.002 | 0.000 | (11; 9) |
| *Diomedea amsterdamensis* | Amsterdam Albatross | Pr | 100 | 135 | 0.001 | 0.000 | (2; 11; 9) |
| *Diomedea antipodensis* | Antipodean Albatross | Pr | 44,500 | 48,950 | 0.510 | 0.085 | (2; 11; 9) |
| *Diomedea dabbenena* | Tristan Albatross | Pr | 4,700 | 5,476 | 0.030 | 0.005 | (2; 11; 9) |
| *Diomedea epomophora* | Southern Royal Albatross | Pr | 27,200 | 35,632 | 0.228 | 0.038 | (2; 11; 9) |
| *Diomedea exulans* | Wandering Albatross | Pr | 20,100 | 23,417 | 0.150 | 0.025 | (2; 11; 9) |
| *Diomedea sanfordi* | Northern Royal Albatross | Pr | 17,000 | 19,635 | 0.175 | 0.029 | (2; 11; 9) |
| *Eudyptes chrysocome* | Rockhopper Penguin | S | 1,750,000 | 2,126,249 | 7.164 | 1.194 | (2; 9) |
| *Eudyptes chrysolophus* | Macaroni Penguin | S | 18,900,000 | 23,058,000 | 107.580 | 17.930 | (2; 9) |
| *Eudyptes pachyrhynchus* | Fiordland Penguin | S | 5,500 | 8,250 | 0.036 | 0.006 | (2; 9) |
| *Eudyptes robustus* | Snares Penguin | S | 135,000 | 178,200 | 0.796 | 0.133 | (2; 9) |
| *Eudyptes schlegeli* | Royal Penguin | S | 62,000 | 75,640 | 0.429 | 0.072 | (2; 9) |
| *Eudyptes sclateri* | Erect-crested Penguin | S | 1,700,000 | 2,244,000 | 10.879 | 1.813 | (2; 9) |
| *Eudyptula minor* | Little Penguin | S | 666,667 | 783,333 | 0.971 | 0.162 | (2; 10; 9) |
| *Fratercula arctica* | Atlantic Puffin | C | 5,850,000 | 7,926,750 | 1.848 | 0.308 | (2; 29; 9) |
| *Fratercula cirrhata* | Tufted Puffin | C | 3,500,000 | 4,462,500 | 1.428 | 0.238 | (2; 11; 9) |
| *Fratercula corniculata* | Horned Puffin | C | 1,200,000 | 1,608,000 | 0.488 | 0.081 | (2; 11; 9) |
| *Fregata andrewsi* | Christmas Island Frigatebird | Pe | 3,600 | 3,960 | 0.009 | 0.001 | (2; 18; 9) |
| *Fregata aquila* | Ascension Frigatebird | Pe | 19,000 | 20,615 | 0.050 | 0.008 | (2; 11; 9) |
| *Fregata ariel* | Lesser Frigatebird | Pe | 200,000 | 220,000 | 0.299 | 0.050 | (2; 11; 9) |
| *Fregata magnificens* | Magnificent Frigatebird | Pe | 40,000 | 44,000 | 0.105 | 0.018 | (2; 11; 9) |
| *Fregata minor* | Great Frigatebird | Pe | 750,000 | 881,250 | 1.588 | 0.265 | (2; 11; 9) |
| *Fregetta grallaria* | White-bellied Storm-petrel | Pr | 300,000 | 375,000 | 0.005 | 0.001 | (2; 11; 9) |
| *Fregetta maoriana* | New Zealand Storm-petrel | Pr | 25 | 31 | 0.000 | 0.000 | (2; 4; 9) |
| *Fregetta tropica* | Black-bellied Storm-petrel | Pr | 500,000 | 625,000 | 0.008 | 0.001 | (2; 4; 9) |
| *Fulmarus glacialis* | Northern Fulmar | Pr | 22,500,000 | 28,912,500 | 26.864 | 4.477 | (2; 9) |
| *Fulmarus glacialoides* | Southern Fulmar | Pr | 4,000,000 | 5,140,000 | 2.891 | 0.482 | (2; 4; 9) |
| *Garrodia nereis* | Grey-backed Storm-petrel | Pr | 200,000 | 250,000 | 0.002 | 0.000 | (2; 4; 9) |
| *Gygis alba* | Common White Tern | C | 617,500 | 790,400 | 0.118 | 0.020 | (2; 31; 9) |
| *Gygis microrhyncha* | Little White Tern | C | 10,000 | 12,800 | 0.002 | 0.000 | (2; 9) |
| *Halobaena caerulea* | Blue Petrel | Pr | 3,000,000 | 3,750,000 | 0.358 | 0.060 | (2; 4; 9) |
| *Hydrobates castro* | Madeiran Storm-petrel | Pr | 150,000 | 180,000 | 0.003 | 0.001 | (2; 4; 9) |
| *Hydrobates furcatus* | Fork Tailed Storm-Petrel | Pr | 6,000,000 | 8,100,000 | 0.088 | 0.015 | (2; 4; 9) |
| *Hydrobates homochroa* | Ashy Storm-Petrel | Pr | 7,650 | 9,945 | 0.000 | 0.000 | (2; 4; 9) |
| *Hydrobates hornbyi* | Hornby's Storm-petrel | Pr | 45,503 | 55,741 | 0.001 | 0.000 | (2; 4; 9) |
| *Hydrobates leucorhous* | Leach's Storm-petrel | Pr | 20,000,000 | 25,000,000 | 0.351 | 0.058 | (2; 4; 9) |
| *Hydrobates markhami* | Markham's Storm-petrel | Pr | 50,000 | 61,250 | 0.001 | 0.000 | (2; 4; 9) |
| *Hydrobates matsudairae* | Matsudaira's Storm-petrel | Pr | 20,000 | 24,500 | 0.000 | 0.000 | (2; 4; 9) |
| *Hydrobates melania* | Black Storm-Petrel | Pr | 500,000 | 650,000 | 0.009 | 0.001 | (2; 4; 9) |
| *Hydrobates microsoma* | Least Storm Petrel | Pr | 565,000 | 706,250 | 0.005 | 0.001 | (2; 11; 9) |
| *Hydrobates monorhis* | Swinhoe's Storm-petrel | Pr | 260,000 | 338,000 | 0.005 | 0.001 | (2; 4; 9) |
| *Hydrobates monteiroi* | Monteiro's Storm-petrel | Pr | 625 | 749 | 0.000 | 0.000 | (2; 4; 9) |
| *Hydrobates pelagicus* | European Storm-petrel | Pr | 1,500,000 | 1,875,000 | 0.015 | 0.003 | (2; 4; 9) |
| *Hydrobates tethys* | Wedge-rumped Storm-petrel | Pr | 500,000 | 600,000 | 0.005 | 0.001 | (2; 4; 9) |
| *Hydrocoloeus minutus* | Little Gull | C | 183,500 | 330,300 | 0.040 | 0.007 | (2; 31; 9) |
| *Hydroprogne caspia* | Caspian Tern | C | 330,000 | 462,000 | 0.242 | 0.040 | (2; 31; 9) |
| *Larosterna inca* | Inca Tern | C | 150,000 | 180,000 | 0.039 | 0.006 | (2; 9) |
| *Larus argentatus* | Herring Gull | C | 2,100,000 | 3,034,500 | 2.612 | 0.435 | (2; 31; 9) |
| *Larus atlanticus* | Olrog's Gull | C | 12,700 | 18,415 | 0.015 | 0.003 | (2; 28; 32; 9) |
| *Larus atricilla* | Laughing Gull | C | 858,550 | 1,287,825 | 0.416 | 0.069 | (2; 31; 9) |
| *Larus audouinii* | Audouin's Gull | C | 43,600 | 63,220 | 0.045 | 0.008 | (2; 9) |
| *Larus belcheri* | Belcher's Gull | C | 7,370 | 11,055 | 0.006 | 0.001 | (2; 9) |
| *Larus brunnicephalus* | Brown-headed Gull | C | 150,000 | 217,500 | 0.076 | 0.013 | (2; 31; 9) |
| *Larus bulleri* | Black-billed Gull | C | 96,000 | 148,800 | 0.048 | 0.008 | (2; 9) |
| *Larus cachinnans* | Yellow-legged Gull | C | 366,667 | 513,333 | 0.490 | 0.082 | (2; 31; 9) |
| *Larus californicus* | California Gull | C | 621,000 | 931,500 | 0.183 | 0.031 | (2; 31; 9) |
| *Larus canus* | Common Gull | C | 3,100,000 | 4,371,000 | 1.945 | 0.324 | (2; 31; 9) |
| *Larus cirrocephalus* | Grey-headed Gull | C | 697,500 | 1,011,375 | 0.353 | 0.059 | (2; 31; 9) |
| *Larus crassirostris* | Black-tailed Gull | C | 1,100,000 | 1,540,000 | 0.843 | 0.140 | (2; 31; 9) |
| *Larus delawarensis* | Ring-billed Gull | C | 2,550,000 | 3,825,000 | 1.661 | 0.277 | (2; 31; 9) |
| *Larus dominicanus* | Kelp Gull | C | 3,800,000 | 5,890,000 | 4.914 | 0.819 | (2; 9) |
| *Larus fuliginosus* | Lava Gull | C | 700 | 1,225 | 0.000 | 0.000 | (2; 31; 9) |
| *Larus fuscus* | Lesser Black-backed Gull | C | 1,000,667 | 1,305,870 | 1.056 | 0.176 | (2; 31; 9) |
| *Larus genei* | Slender-billed Gull | C | 345,000 | 517,500 | 0.158 | 0.026 | (2; 31; 9) |
| *Larus glaucescens* | Glaucous-winged Gull | C | 570,000 | 883,500 | 1.047 | 0.174 | (2; 31; 9) |
| *Larus glaucoides* | Iceland Gull | C | 295,000 | 457,250 | 0.311 | 0.052 | (2; 31; 9) |
| *Larus hartlaubii* | King Gull | C | 30,000 | 45,000 | 0.015 | 0.002 | (2; 31; 9) |
| *Larus heermanni* | Heermann's Gull | C | 525,000 | 708,750 | 0.382 | 0.064 | (2; 22; 9) |
| *Larus hemprichii* | Sooty Gull | C | 225,000 | 337,500 | 0.126 | 0.021 | (2; 31; 9) |
| *Larus hyperboreus* | Glaucous Gull | C | 1,370,000 | 2,055,000 | 2.056 | 0.343 | (2; 31; 9) |
| *Larus ichthyaetus* | Pallas's Gull | C | 612,500 | 1,041,250 | 1.140 | 0.190 | (2; 31; 9) |
| *Larus leucophthalmus* | White-eyed Gull | C | 40,500 | 60,750 | 0.023 | 0.004 | (2; 31; 9) |
| *Larus livens* | Yellow-footed Gull | C | 60,000 | 75,000 | 0.073 | 0.012 | (2; 31; 9) |
| *Larus maculipennis* | Brown-hooded Gull | C | 556,000 | 834,000 | 0.273 | 0.045 | (2; 31; 9) |
| *Larus marinus* | Great Black-backed Gull | C | 596,667 | 981,517 | 1.161 | 0.193 | (2; 31; 9) |
| *Larus melanocephalus* | Mediterranean Gull | C | 56,667 | 82,167 | 0.023 | 0.004 | (2; 31; 9) |
| *Larus modestus* | Gray Gull | C | 25,000 | 47,500 | 0.015 | 0.003 | (2; 31; 9) |
| *Larus novaehollandiae* | Silver Gull | C | 550,000 | 770,000 | 0.304 | 0.051 | (2; 31; 9) |
| *Larus occidentalis* | Western Gull | C | 117,000 | 152,100 | 0.167 | 0.028 | (2; 31; 9) |
| *Larus pacificus* | Pacific Gull | C | 10,950 | 16,425 | 0.014 | 0.002 | (2; 31; 9) |
| *Larus philadelphia* | Bonaparte's Gull | C | 390,000 | 585,000 | 0.141 | 0.023 | (2; 31; 9) |
| *Larus pipixcan* | Franklin's Gull | C | 830,000 | 1,245,000 | 0.387 | 0.065 | (2; 31; 9) |
| *Larus relictus* | Relict Gull | C | 15,000 | 22,500 | 0.007 | 0.001 | (2; 9) |
| *Larus ridibundus* | Black-headed Gull | C | 6,850,000 | 9,932,500 | 3.027 | 0.505 | (2; 31; 9) |
| *Larus schistisagus* | Slaty-backed Gull | C | 512,500 | 768,750 | 0.853 | 0.142 | (2; 31; 9) |
| *Larus scopulinus* | Red-billed Gull | C | 550,000 | 852,500 | 0.276 | 0.046 | (31; 9) |
| *Larus scoresbii* | Dolphin Gull | C | 19,275 | 27,563 | 0.014 | 0.002 | (2; 31; 9) |
| *Larus serranus* | Andean Gull | C | 100,000 | 145,000 | 0.062 | 0.010 | (2; 31; 9) |
| *Larus thayeri* | Thayer's Gull | C | 17,500 | 26,250 | 0.024 | 0.004 | (2; 31; 9) |
| *Macronectes giganteus* | Southern Giant-petrel | Pr | 82,500 | 118,800 | 0.396 | 0.066 | (2; 9) |
| *Macronectes halli* | Northern Giant-petrel | Pr | 18,750 | 27,188 | 0.064 | 0.011 | (2; 9) |
| *Megadyptes antipodes* | Yellow-eyed Penguin | S | 3,850 | 5,390 | 0.027 | 0.004 | (2; 9) |
| *Microcarbo africanus* | Reed Cormorant | Pe | 467,667 | 537,817 | 0.440 | 0.073 | (2; 31; 9) |
| *Microcarbo coronatus* | Crowned Cormorant | Pe | 8,700 | 14,573 | 0.012 | 0.002 | (2; 9) |
| *Microcarbo melanoleucos* | Little Pied Cormorant | Pe | 79,000 | 150,100 | 0.131 | 0.022 | (2; 31; 9) |
| *Microcarbo niger* | Little Cormorant | Pe | 208,333 | 468,750 | 0.201 | 0.034 | (2; 31; 9) |
| *Microcarbo pygmaeus* | Pygmy Cormorant | Pe | 88,333 | 229,667 | 0.084 | 0.014 | (2; 31; 9) |
| *Morus bassanus* | Northern Gannet | Pe | 1,075,000 | 1,467,375 | 5.097 | 0.850 | (2; 11; 9) |
| *Morus capensis* | Cape Gannet | Pe | 300,000 | 414,000 | 1.339 | 0.223 | (2; 11; 9) |
| *Morus serrator* | Australasian Gannet | Pe | 214,133 | 281,585 | 0.629 | 0.105 | (2; 6; 9) |
| *Nesofregetta fuliginosa* | White-throated Storm-petrel | Pr | 625 | 781 | 0.000 | 0.000 | (2; 4; 9) |
| *Oceanites gracilis* | White-vented Storm-petrel | Pr | 30,000 | 36,750 | 0.000 | 0.000 | (2; 4; 9) |
| *Oceanites oceanicus* | Wilson's Storm-petrel | Pr | 21,000,000 | 25,200,000 | 0.380 | 0.063 | (2; 4; 9) |
| *Oceanodroma tristrami* | Tristram’s Storm-Petrel | Pr | 20,000 | 24,500 | 0.000 | 0.000 | (2; 4; 9) |
| *Onychoprion aleuticus* | Aleutian Tern | C | 32,500 | 46,313 | 0.006 | 0.001 | (2; 31; 9) |
| *Onychoprion anaethetus* | Bridled Tern | C | 1,055,000 | 1,482,275 | 0.231 | 0.039 | (2; 31; 9) |
| *Onychoprion fuscatus* | Sooty Tern | C | 21,500,000 | 26,875,000 | 5.879 | 0.980 | (2; 31; 9) |
| *Onychoprion lunatus* | Grey-backed Tern | C | 550,000 | 687,500 | 0.089 | 0.015 | (2; 31; 9) |
| *Pachyptila belcheri* | Slender-billed Prion | Pr | 7,000,000 | 8,330,000 | 0.799 | 0.133 | (2; 4; 9) |
| *Pachyptila crassirostris* | Fulmar Prion | Pr | 225,000 | 281,250 | 0.023 | 0.004 | (2; 4; 9) |
| *Pachyptila desolata* | Antarctic Prion | Pr | 50,000,000 | 62,500,000 | 5.151 | 0.858 | (2; 4; 9) |
| *Pachyptila salvini* | Medium-billed Prion | Pr | 12,000,000 | 15,000,000 | 1.382 | 0.230 | (2; 4; 9) |
| *Pachyptila turtur* | Fairy Prion | Pr | 5,000,000 | 6,250,000 | 0.517 | 0.086 | (2; 4; 9) |
| *Pachyptila vittata* | Broad-billed Prion | Pr | 15,000,000 | 18,750,000 | 2.075 | 0.346 | (2; 4; 9) |
| *Pagodroma nivea* | Snow Petrel | Pr | 4,000,000 | 5,000,000 | 1.035 | 0.173 | (2; 4; 9) |
| *Pagophila eburnea* | Ivory Gull | C | 15,000 | 22,500 | 0.009 | 0.002 | (2; 31; 9) |
| *Papasula abbotti* | Abbott's Booby | Pe | 6,000 | 6,600 | 0.019 | 0.003 | (2; 11; 9) |
| *Pelagodroma marina* | White-faced Storm-petrel | Pr | 4,000,000 | 5,200,000 | 0.068 | 0.011 | (2; 4; 9) |
| *Pelecanoides georgicus* | South Georgia Diving-petrel | Pr | 15,000,000 | 20,250,000 | 1.480 | 0.247 | (2; 4; 9) |
| *Pelecanoides urinatrix* | Common Diving-petrel | Pr | 16,000,000 | 22,400,000 | 1.622 | 0.270 | (2; 4; 9) |
| *Pelecanus occidentalis* | Brown Pelican | Pe | 248,110 | 396,976 | 1.595 | 0.266 | (2; 11; 9) |
| *Phaethon aethereus* | Red-billed Tropicbird | Pe | 12,225 | 15,037 | 0.011 | 0.002 | (2; 11; 9) |
| *Phaethon lepturus* | White-tailed Tropicbird | Pe | 50,000 | 60,750 | 0.041 | 0.007 | (2; 11; 9) |
| *Phaethon rubricauda* | Red-tailed Tropicbird | Pe | 32,000 | 39,680 | 0.035 | 0.006 | (2; 11; 9) |
| *Phaetusa simplex* | Large-billed Tern | C | 53,333 | 69,333 | 0.018 | 0.003 | (2; 31; 9) |
| *Phalacrocorax aristotelis* | European shag | Pe | 237,000 | 395,790 | 0.765 | 0.127 | (2; 31; 9) |
| *Phalacrocorax atriceps* | Imperial Shag | Pe | 844,165 | 1,435,081 | 2.929 | 0.488 | (2; 31; 9) |
| *Phalacrocorax auritus* | Double-crested Cormorant | Pe | 746,290 | 1,291,082 | 2.725 | 0.454 | (2; 31; 9) |
| *Phalacrocorax bougainvilliorum* | Guanay Cormorant | Pe | 3,750,000 | 8,249,999 | 14.377 | 2.396 | (2; 9) |
| *Phalacrocorax brasilianus* | Neotropic Cormorant | Pe | 2,000,000 | 3,650,000 | 2.803 | 0.467 | (2; 9) |
| *Phalacrocorax campbelli* | Campbell Island Shag | Pe | 8,000 | 9,600 | 0.018 | 0.003 | (2; 31; 9) |
| *Phalacrocorax capensis* | Cape Cormorant | Pe | 190,000 | 318,250 | 0.402 | 0.067 | (2; 9) |
| *Phalacrocorax capillatus* | Japanese Cormorant | Pe | 62,500 | 137,500 | 0.314 | 0.052 | (2; 31; 9) |
| *Phalacrocorax carbo* | Great Cormorant | Pe | 2,150,000 | 4,472,000 | 6.734 | 1.122 | (2; 31; 9) |
| *Phalacrocorax carunculatus* | King Shag | Pe | 625 | 781 | 0.002 | 0.000 | (2; 9) |
| *Phalacrocorax chalconotus* | Stewart Island Shag | Pe | 6,450 | 7,740 | 0.024 | 0.004 | (2; 9) |
| *Phalacrocorax colensoi* | Auckland Shag | Pe | 3,000 | 3,600 | 0.007 | 0.001 | (2; 9) |
| *Phalacrocorax featherstoni* | Pitt Cormorant | Pe | 1,094 | 1,313 | 0.003 | 0.001 | (2; 11; 9) |
| *Phalacrocorax fuscescens* | Black-faced Shag | Pe | 6,667 | 11,167 | 0.015 | 0.002 | (2; 11; 9) |
| *Phalacrocorax fuscicollis* | Indian Cormorant | Pe | 30,000 | 67,500 | 0.029 | 0.005 | (2; 31; 9) |
| *Phalacrocorax gaimardi* | Red-legged Cormorant | Pe | 30,000 | 66,000 | 0.078 | 0.013 | (2; 9) |
| *Phalacrocorax harrisi* | Flightless Cormorant | Pe | 1,338 | 3,011 | 0.014 | 0.002 | (2; 9) |
| *Phalacrocorax magellanicus* | Rock Shag | Pe | 102,667 | 205,333 | 0.377 | 0.063 | (2; 31; 9) |
| *Phalacrocorax neglectus* | Bank Cormorant | Pe | 5,600 | 9,800 | 0.017 | 0.003 | (2; 9) |
| *Phalacrocorax nigrogularis* | Socotra Cormorant | Pe | 220,000 | 368,500 | 0.771 | 0.129 | (2; 19; 9) |
| *Phalacrocorax onslowi* | Chatham Island Shag | Pe | 720 | 1,080 | 0.002 | 0.000 | (2; 9) |
| *Phalacrocorax pelagicus* | Pelagic Cormorant | Pe | 11,667 | 24,500 | 0.041 | 0.007 | (2; 31; 9) |
| *Phalacrocorax penicillatus* | Brandt's Cormorant | Pe | 66,667 | 130,000 | 0.238 | 0.040 | (2; 31; 9) |
| *Phalacrocorax punctatus* | Spotted Shag | Pe | 92,500 | 187,313 | 0.265 | 0.044 | (2; 31; 9) |
| *Phalacrocorax ranfurlyi* | Bounty Islands Shag | Pe | 615 | 1,076 | 0.002 | 0.000 | (2; 9) |
| *Phalacrocorax sulcirostris* | Little Black Shag | Pe | 353,333 | 678,400 | 0.266 | 0.044 | (2; 31; 9) |
| *Phalacrocorax urile* | Red-faced Cormorant | Pe | 200,000 | 325,000 | 0.612 | 0.102 | (2; 31; 9) |
| *Phalacrocorax varius* | Greater Pied Cormorant | Pe | 53,333 | 89,067 | 0.172 | 0.029 | (2; 31; 9) |
| *Phalacrocorax verrucosus* | Kerguelen Shag | Pe | 4,333 | 7,367 | 0.015 | 0.003 | (2; 11; 9) |
| *Phoebastria albatrus* | Short-tailed Albatross | Pr | 2,400 | 3,012 | 0.014 | 0.002 | (2; 11; 9) |
| *Phoebastria immutabilis* | Laysan Albatross | Pr | 1,180,000 | 1,463,200 | 6.181 | 1.030 | (2; 11; 9) |
| *Phoebastria irrorata* | Waved Albatross | Pr | 34,700 | 39,038 | 0.197 | 0.033 | (2; 11; 9) |
| *Phoebastria nigripes* | Black-footed Albatross | Pr | 129,000 | 161,250 | 0.671 | 0.112 | (2; 11; 9) |
| *Phoebetria fusca* | Sooty Albatross | Pr | 42,000 | 46,620 | 0.083 | 0.014 | (2; 11; 9) |
| *Phoebetria palpebrata* | Light-mantled Albatross | Pr | 58,000 | 76,270 | 0.314 | 0.052 | (2; 11; 9) |
| *Procellaria aequinoctialis* | White-chinned Petrel | Pr | 3,000,000 | 3,735,000 | 1.836 | 0.306 | (2; 4; 9) |
| *Procellaria cinerea* | Grey Petrel | Pr | 400,000 | 540,000 | 0.209 | 0.035 | (2; 4; 9) |
| *Procellaria conspicillata* | Spectacled Petrel | Pr | 30,000 | 36,750 | 0.006 | 0.001 | (2; 9) |
| *Procellaria parkinsoni* | Parkinson's Petrel | Pr | 4,950 | 6,064 | 0.002 | 0.000 | (2; 9) |
| *Procellaria westlandica* | Westland Petrel | Pr | 10,700 | 13,108 | 0.007 | 0.001 | (2; 9) |
| *Procelsterna cerulea* | Blue Noddy | C | 73,500 | 91,875 | 0.006 | 0.001 | (2; 9) |
| *Pseudobulweria aterrima* | Mascarene Petrel | Pr | 150 | 184 | 0.000 | 0.000 | (2; 9) |
| *Pseudobulweria becki* | Beck's Petrel | Pr | 150 | 183 | 0.000 | 0.000 | (2; 9) |
| *Pseudobulweria macgillivrayi* | Fiji Petrel | Pr | 25 | 31 | 0.000 | 0.000 | (2; 9) |
| *Pseudobulweria rostrata* | Tahiti Petrel | Pr | 15,000 | 18,749 | 0.002 | 0.000 | (2; 9) |
| *Pterodrom leucoptera* | Gould's Petrel | Pr | 12,000 | 14,700 | 0.002 | 0.000 | (2; 4; 9) |
| *Pterodroma alba* | Phoenix Petrel | Pr | 30,000 | 37,500 | 0.003 | 0.001 | (2; 9) |
| *Pterodroma arminjoniana* | Trindade Petrel | Pr | 8,050 | 9,861 | 0.004 | 0.001 | (2; 23; 9) |
| *Pterodroma atrata* | Henderson Petrel | Pr | 32,000 | 35,200 | 0.017 | 0.003 | (2; 4 and 5; 9) |
| *Pterodroma axillaris* | Chatham Petrel | Pr | 1,100 | 1,348 | 0.000 | 0.000 | (2; 4; 9) |
| *Pterodroma baraui* | Barau's Petrel | Pr | 7,000 | 7,700 | 0.003 | 0.000 | (2; 4; 9) |
| *Pterodroma brevipes* | Collared Petrel | Pr | 5,528 | 6,081 | 0.001 | 0.000 | (2; 4; 9) |
| *Pterodroma cahow* | Bermuda Petrel | Pr | 142 | 160 | 0.000 | 0.000 | (2; 4; 9) |
| *Pterodroma cookii* | Cook's Petrel | Pr | 1,005,000 | 1,206,000 | 0.141 | 0.024 | (2; 4; 9) |
| *Pterodroma defilippiana* | De Filippi's Petrel | Pr | 15,750 | 19,294 | 0.002 | 0.000 | (2; 4; 9) |
| *Pterodroma externa* | Juan Fernandez Petrel | Pr | 2,000,000 | 2,250,000 | 0.782 | 0.130 | (2; 4; 9) |
| *Pterodroma feae* | Fea's Petrel | Pr | 1,500 | 1,650 | 0.000 | 0.000 | (2; 4; 9) |
| *Pterodroma hasitata* | Black-capped Petrel | Pr | 5,000 | 6,125 | 0.001 | 0.000 | (2; 4; 9) |
| *Pterodroma heraldica* | Herald Petrel | Pr | 150,000 | 172,500 | 0.030 | 0.005 | (2; 4; 9) |
| *Pterodroma hypoleuca* | Bonin Petrel | Pr | 666,667 | 750,000 | 0.079 | 0.013 | (2; 4; 9) |
| *Pterodroma incerta* | Atlantic Petrel | Pr | 5,000,000 | 5,500,000 | 1.482 | 0.247 | (2; 9) |
| *Pterodroma inexpectata* | Mottled Petrel | Pr | 320,000 | 424,000 | 0.090 | 0.015 | (2; 26; 9) |
| *Pterodroma lessonii* | White-headed Petrel | Pr | 600,000 | 645,000 | 0.397 | 0.066 | (2; 4; 9) |
| *Pterodroma longirostris* | Stejneger's Petrel | Pr | 400,000 | 500,000 | 0.067 | 0.011 | (2; 9) |
| *Pterodroma macroptera* | Great-winged Petrel | Pr | 1,500,000 | 1,650,000 | 0.432 | 0.072 | (2; 4; 9) |
| *Pterodroma magentae* | Magenta Petrel | Pr | 135 | 165 | 0.000 | 0.000 | (2; 9) |
| *Pterodroma mollis* | Soft-plumaged Petrel | Pr | 5,000,000 | 6,000,000 | 1.008 | 0.168 | (2; 4; 9) |
| *Pterodroma neglecta* | Kermadec Petrel | Pr | 175,000 | 183,750 | 0.100 | 0.017 | (2; 4; 9) |
| *Pterodroma nigripennis* | Black-winged Petrel | Pr | 9,000,000 | 12,375,000 | 2.406 | 0.401 | (2; 4; 9) |
| *Pterodroma phaeopygia* | Galapagos Petrel | Pr | 15,750 | 17,640 | 0.004 | 0.001 | (2; 9) |
| *Pterodroma pycrofti* | Pycroft's Petrel | Pr | 17,000 | 21,250 | 0.002 | 0.000 | (2; 9) |
| *Pterodroma sandwichensis* | Hawaiian Petrel | Pr | 12,750 | 15,938 | 0.009 | 0.001 | (2; 4; 9) |
| *Pterodroma solandri* | Providence Petrel | Pr | 100,000 | 117,500 | 0.025 | 0.004 | (2; 9) |
| *Pterodroma ultima* | Murphy's Petrel | Pr | 862,500 | 927,188 | 0.432 | 0.072 | (2; 4; 9) |
| *Ptychoramphus aleuticus* | Cassin's Auklet | C | 3,250,000 | 4,225,000 | 0.515 | 0.086 | (2; 16; 9) |
| *Puffinus assimilis* | Little Shearwater | Pr | 300,000 | 367,499 | 0.023 | 0.004 | (2; 9) |
| *Puffinus auricularis* | Newell’s Shearwater | Pr | 625 | 765 | 0.000 | 0.000 | (2; 9) |
| *Puffinus gavia* | Fluttering Shearwater | Pr | 100,000 | 122,500 | 0.011 | 0.002 | (2; 4; 9) |
| *Puffinus heinrothi* | Heinroth's Shearwater | Pr | 625 | 809 | 0.000 | 0.000 | (2; 9) |
| *Puffinus huttoni* | Hutton's Shearwater | Pr | 325,000 | 390,000 | 0.036 | 0.006 | (2; 4; 9) |
| *Puffinus lherminieri* | Audubon’s Shearwater | Pr | 35,000 | 45,324 | 0.002 | 0.000 | (2; 4; 9) |
| *Puffinus mauretanicus* | Balearic Shearwater | Pr | 11,000 | 14,025 | 0.002 | 0.000 | (2; 9) |
| *Puffinus nativitatis* | Christmas Shearwater | Pr | 150,000 | 183,750 | 0.066 | 0.011 | (2; 4; 9) |
| *Puffinus opisthomelas* | Black-vented Shearwater | Pr | 160,000 | 196,000 | 0.015 | 0.002 | (2; 9) |
| *Puffinus Puffinus* | Manx Shearwater | Pr | 1,000,000 | 1,350,000 | 0.097 | 0.016 | (2; 4; 9) |
| *Puffinus yelkouan* | Yelkouan Shearwater | Pr | 46,000 | 61,410 | 0.004 | 0.001 | (2; 9) |
| *Pygoscelis adeliae* | Adelie Penguin | S | 4,740,000 | 7,157,400 | 29.232 | 4.872 | (2; 9) |
| *Pygoscelis antarcticus* | Chinstrap Penguin | S | 8,000,000 | 12,800,000 | 55.551 | 9.259 | (2; 9) |
| *Pygoscelis papua* | Gentoo Penguin | S | 774,000 | 1,161,000 | 5.272 | 0.879 | (2; 9) |
| *Rhodostethia rosea* | Ross's Gull | C | 62,500 | 93,750 | 0.022 | 0.004 | (2; 31; 9) |
| *Rissa brevirostris* | Red-legged Kittiwake | C | 357,000 | 401,625 | 0.253 | 0.042 | (2; 9) |
| *Rissa tridactyla* | Black-legged Kittiwake | C | 17,500,000 | 24,325,000 | 10.531 | 1.755 | (2; 31; 9) |
| *Saundersilarus saundersi* | Saunders's Gull | C | 14,400 | 20,880 | 0.006 | 0.001 | (1; 9) |
| *Spheniscus demersus* | African Penguin | S | 52,000 | 67,860 | 0.157 | 0.026 | (2; 9) |
| *Spheniscus humboldti* | Humboldt penguin | S | 6,250 | 8,437 | 0.026 | 0.004 | (2; 9) |
| *Spheniscus magellanicus* | Magellanic Penguin | S | 2,600,000 | 3,328,000 | 6.842 | 1.140 | (2; 9) |
| *Spheniscus mendiculus* | Galapagos Penguin | S | 1,800 | 3,150 | 0.006 | 0.001 | (2; 9) |
| *Stercorarius longicaudus* | Long-tailed Skua | C | 2,575,000 | 2,896,875 | 1.377 | 0.230 | (2; 9) |
| *Stercorarius parasiticus* | Parasitic Skua | C | 5,250,000 | 6,536,250 | 3.504 | 0.584 | (2; 11; 9) |
| *Stercorarius pomarinus* | Pomarine Skua | C | 1,625,000 | 2,258,750 | 2.664 | 0.444 | (2; 11; 9) |
| *Sterna acuticauda* | Black-bellied Tern | C | 11,850 | 17,301 | 0.002 | 0.000 | (2; 9) |
| *Sterna aurantia* | River Tern | C | 50,000 | 58,750 | 0.017 | 0.003 | (2; 12; 9) |
| *Sterna dougallii* | Roseate Tern | C | 76,000 | 132,620 | 0.011 | 0.002 | (2; 9) |
| *Sterna forsteri* | Forster's Tern | C | 48,783 | 65,858 | 0.011 | 0.002 | (2; 9) |
| *Sterna hirundinacea* | South American Tern | C | 512,500 | 602,188 | 0.139 | 0.023 | (2; 31; 9) |
| *Sterna hirundo* | Common Tern | C | 2,066,667 | 3,358,333 | 0.417 | 0.070 | (2; 31; 9) |
| *Sterna nilotica* | Gull-billed tern | C | 285,000 | 495,900 | 0.077 | 0.013 | (2; 31; 9) |
| *Sterna paradisaea* | Arctic Tern | C | 2,000,000 | 2,800,000 | 0.337 | 0.056 | (2; 31; 9) |
| *Sterna repressa* | White-cheeked Tern | C | 600,000 | 876,000 | 0.117 | 0.020 | (2; 31; 9) |
| *Sterna striata* | White-fronted Tern | C | 27,000 | 37,800 | 0.008 | 0.001 | (2; 31; 9) |
| *Sterna sumatrana* | Black-naped Tern | C | 92,367 | 101,603 | 0.015 | 0.003 | (2; 9) |
| *Sterna superciliaris* | Yellow-billed Tern | C | 41,667 | 55,417 | 0.004 | 0.001 | (2; 31; 9) |
| *Sterna trudeaui* | Snowy-crowned Tern | C | 5,500 | 7,150 | 0.001 | 0.000 | (2; 11; 9) |
| *Sterna virgata* | Kerguelen Tern | C | 4,950 | 5,544 | 0.001 | 0.000 | (2; 9) |
| *Sterna vittata* | Antarctic Tern | C | 92,483 | 118,379 | 0.020 | 0.003 | (2; 31; 9) |
| *Sternula albifrons* | Little Tern | C | 300,000 | 585,000 | 0.026 | 0.004 | (2; 31; 9) |
| *Sternula antillarum* | Least Tern | C | 53,267 | 70,578 | 0.004 | 0.001 | (2; 31; 9) |
| *Sternula balaenarum* | Damara Tern | C | 13,950 | 17,856 | 0.001 | 0.000 | (2; 11; 9) |
| *Sternula lorata* | Peruvian Tern | C | 1,725 | 2,070 | 0.000 | 0.000 | (2; 9) |
| *Sternula nereis* | Fairy Tern | C | 6,250 | 6,874 | 0.001 | 0.000 | (2; 31; 9) |
| *Sternula saundersi* | Saunders's Tern | C | 26,667 | 38,000 | 0.002 | 0.000 | (2; 31; 9) |
| *Sula dactylatra* | Masked Booby | Pe | 90,000 | 112,950 | 0.342 | 0.057 | (2; 11; 9) |
| *Sula granti* | Nazca Booby | Pe | 20,000 | 25,000 | 0.032 | 0.005 | (2; 9) |
| *Sula leucogaster* | Brown Booby | Pe | 200,000 | 280,000 | 0.446 | 0.074 | (2; 11; 9) |
| *Sula nebouxii* | Blue-footed Booby | Pe | 80,000 | 98,000 | 0.243 | 0.040 | (2; 17; 9) |
| *Sula sula* | Red-footed Booby | Pe | 1,000,000 | 1,200,000 | 1.960 | 0.327 | (2; 11; 9) |
| *Sula variegata* | Peruvian booby | Pe | 1,160,000 | 1,421,000 | 2.613 | 0.435 | (2; 11; 9) |
| *Synthliboramphus antiquus* | Ancient Murrelet | C | 1,500,000 | 2,655,000 | 0.156 | 0.026 | (2; 11; 9) |
| *Synthliboramphus craveri* | Craveri's Murrelet | C | 12,000 | 18,000 | 0.001 | 0.000 | (2; 14; 9) |
| *Synthliboramphus hypoleucus* | Xantus's Murrelet | C | 5,000 | 6,800 | 0.001 | 0.000 | (2; 3; 9) |
| *Synthliboramphus wumizusume* | Japanese Murrelet | C | 6,250 | 7,374 | 0.002 | 0.000 | (2; 9) |
| *Thalassarche bulleri* | Buller's Albatross | Pr | 64,000 | 82,560 | 0.270 | 0.045 | (2; 11; 9) |
| *Thalassarche carteri* | Indian Yellow-nosed Albatross | Pr | 83,160 | 93,139 | 0.330 | 0.055 | (2; 11; 9) |
| *Thalassarche cauta* | Shy Albatross | Pr | 30,700 | 38,529 | 0.174 | 0.029 | (2; 11; 9) |
| *Thalassarche chlororhynchos* | Atlantic Yellow-nosed Albatross | Pr | 26,500 | 35,775 | 0.101 | 0.017 | (2; 11; 9) |
| *Thalassarche chrysostoma* | Grey-headed Albatross | Pr | 250,000 | 312,500 | 0.698 | 0.116 | (2; 11; 9) |
| *Thalassarche eremita* | Chatham Albatross | Pr | 11,000 | 15,400 | 0.070 | 0.012 | (2; 11; 9) |
| *Thalassarche impavida* | Campbell Albatross | Pr | 49,200 | 61,500 | 0.224 | 0.037 | (2; 11; 9) |
| *Thalassarche melanophrys* | Black-browed Albatross | Pr | 1,150,000 | 1,443,250 | 6.787 | 1.131 | (2; 11; 9) |
| *Thalassarche salvini* | Salvin's Albatross | Pr | 61,500 | 77,183 | 0.349 | 0.058 | (2; 11; 9) |
| *Thalassarche steadi* | White-capped Albatross | Pr | 300,000 | 375,000 | 1.731 | 0.288 | (2; 11; 9) |
| *Thalasseus bengalensis* | Lesser Crested Tern | C | 183,333 | 256,667 | 0.057 | 0.010 | (2; 31; 9) |
| *Thalasseus bergii* | Greater Crested Tern | C | 416,667 | 489,583 | 0.182 | 0.030 | (2; 9) |
| *Thalasseus bernsteini* | Chinese Crested Tern | C | 40 | 46 | 0.000 | 0.000 | (2; 9) |
| *Thalasseus elegans* | Elegant tern | C | 70,500 | 90,240 | 0.024 | 0.004 | (2; 22; 9) |
| *Thalasseus maximus* | Royal Tern | C | 374,900 | 479,872 | 0.207 | 0.035 | (2; 31; 9) |
| *Thalasseus sandvicensis* | Sandwich Tern | C | 563,400 | 847,917 | 0.191 | 0.032 | (2; 31; 9) |
| *Thalassoica antarctica* | Antarctic Petrel | Pr | 15,000,000 | 18,750,000 | 8.830 | 1.472 | (2; 4; 9) |
| *Uria aalge* | Common Guillemot | C | 18,000,000 | 24,390,000 | 21.564 | 3.594 | (2; 11; 9) |
| *Uria lomvia* | Thick-billed Murre | C | 22,000,000 | 30,800,000 | 21.070 | 3.512 | (2; 11; 9) |
| *Xema sabini* | Sabines Gull | C | 515,000 | 620,575 | 0.174 | 0.029 | (2; 31; 9) |
| **TOTAL** |  |  | **612,993,610** | **804,303,191** | **591.263** | **98.544** |  |

*:Charadriiformes (C); Pelecaniformes (Pe); Procellariiformes (Pr); Sphenisciformes (S)

**: The number of chicks was calculated applying a reproductive efficiency factor for each species, as calculated by Riddick et al.^25^

***: Total excreted N and P refer to breeders and chicks.

**References**

1. BirdLife International (BI). *Threatened birds of Asia: the BI Red Data Book*. (BI, Cambridge, UK 2001).

2. BirdLife International (BI) *IUCN Red List for birds*. <http://www.birdlife.org> (2014).

3. Birt, T. et al. Rangewide population genetic structure of Xantus´s Murrelet (*Synthliboramphus hypoleucus*). *Auk* **129**, 44-55 (2012).

4. Brooke, M. L. *Albatrosses and petrels across the world* (University Press, Oxford, 2004).

5. Brooke, M. L. et al. The potential for rat predation to cause decline of the globally threatened Henderson petrel *Pterodroma atrata*: evidence from population modelling, the field and stable isotopes. *Endanger. Species Res.* **11**, 47-59 (2010).

6. Bunce, A., Norman, F., Brothers, N. & Gales, R. Long-term trends in the Australasian gannet (*Morus serrator*) population in Australia: the effect of climate change and commercial fisheries. *Mar. Biol.* **141**, 263-269 (2002).

7. Castro, D. et al. Aves, Stercorariidae, Chilean Skua *Stercorarius chilensis* Bonaparte, 1857: First documented record for the state of Espírito Santo, Southeastern Brazil. *Check List* **8**, 560-562 (2012).

8. COSEWIC. Update COSEWIC Status Report on the Marbled Murrelet (*Brachyramphus marmoratus*) in Canada. (Committee on the Status of Wildlife In Canada, Ottawa, ON 2012).

9. Croxall, J. P. et al. Seabird conservation status, threats and priority actions: a global assessment. *Bird Conserv. Int.* **22**, 1-34 (2012).

10. del Hoyo, J., Elliot, A. & Sargatal, J. *Handbook of the Birds of the World*, vol. 1: Ostrich to Ducks (Lynx Edicions, Spain, 1992).

11. del Hoyo, J., Elliott, A. & Sargatal, J. *Handbook of the Birds of the World*, vol. 3: Hoatzin to Auks (Lynx Edicions, Spain, 1996).

12. Delany, S. & Scott, D. *Waterbird Population Estimates* (Wetlands International, Wageningen, The Netherlands, 2006).

13. Department of the Environment. *Stercorarius antarcticus lonnbergi* in Species Profile and Threats Database, <http://www.environment.gov.au/sprat> (2014).

14. Encyclopedia of Life. <http://www.eol.org> (2014).

15. Fang, E. D. Snowy Sheathbill (*Chionis albus*). in *Neotropical Birds Online* (ed Schulenberg, T.S.) <http://neotropical.birds.cornell.edu/portal/species/overview> (2010).

16. Harfenist, A. Cassin’s auklet (*Ptychoramphus aleuticus*) (Accounts and Measures for Managing Identified Wildlife-Accounts, British Columbia, Canada, 2004).

17. Harris, M. *Sula nebouxii*, Animal Diversity (<http://animaldiversity.ummz.umich.edu/accounts/Sula_nebouxii/>, 2001).

18. Hill, R. & Dunn, A. National recovery plan for the Christmas Island Frigatebird (*Fregata andrewsi*) (Commonwealth of Australia, Canberra. 2004).

19. Jennings, M. C. Atlas of the breeding birds of Arabia. *Fauna Arabia* **25**, 1-772 (2010).

20. John, R. C. et al. *Ecology and conservation of the Marbled Murrelet*. (Gen. Tech. Rep. PSW-GTR-152, Pacific Southwest Research Station, Forest Service, U.S. Department of Agriculture, 1995).

21. Joint Nature Conservation Committee (JNCC) (<http://www.jncc.defra.gov.uk>, 2014).

22. Kushlan, J. A. et al. *Waterbird conservation for the Americas*. (U.S. Fish & Wildlife Service National Publications Clearinghouse, Shepherdstown, U.S.A., 2002).

23. Luigi, G., Bugoni, L., Fonseca-Neto, F. P. & Teixeira, D. M. Biologia e conservação do petrel-de trindade, *Pterodroma arminjoniana*, na ilha da Trindade, Atlântico sul. In*: Ilhas oceánicas brasileiras: da pesquisa ao manejo* (eds Mohr, L. V., Castro, J. W. A., Costa, P. M. S., Alves, R. J. V.) vol 2 (Ministério do Meio Ambiente, Brasília, Brazil, 2008).

24. Marchant, S. & Higgins, P. J. *Handbook of Australian, New Zealand and Antarctic Birds, 1: ratites to ducks* (Oxford University Press, Oxford, UK,1990).

25. Riddick, S. N. et al. The global distribution of ammonia emissions from seabird colonies. *Atmospheric Environ.* **55**, 319-327 (2012).

26. Scott, D. et. al. Predictive habitat modelling to estimate petrel breeding colony sizes: Sooty shearwaters (*Puffinus griseus*) and mottled petrels (*Pterodroma inexpectata*) on Whenua Hou Island. *N.Z. J. Zool.* **36**, 291-306 (2009).

27. Shirihai, H. A. *Complete Guide to Antarctic Wildlife* (A&C Black, London, UK, 2002).

28. Suárez, N., Retana, M. V. & Yorio, P. Spatial patterns in the use of foraging areas and its relationship with prey resources in the threatened Olrog’s Gull (*Larus atlanticus*). *J. Ornithol.* **153**, 861–871 (2012).

29. The MarineBio Conservation Society. <http://marinebio.org/membership/join> (2014).

30. U.S. Fish and Wildlife Service. *Endangered and Threatened Wildlife and Plants; Review of Native Species That Are Candidates for Listing as Endangered or Threatened*. (<http://www.fws.gov/endangered/what-we-do/cnor.html>, 2010).

31. Wetlands International. *Waterbird Population Estimates*. (4th ed. Wetlands International, Wageningen, The Netherlands, 2006).

32. Yorio, P., Quintana, F., Gatto, A., Lisnizer, N. & Suárez, N. Foraging patterns of breeding Olrog's Gull at Golfo San Jorge, Argentina. *Waterbirds* **27**, 193-199 (2004).
